# Brain-targeted drug delivery by manipulating protein corona functions

**DOI:** 10.1038/s41467-019-11593-z

**Published:** 2019-08-08

**Authors:** Zui Zhang, Juan Guan, Zhuxuan Jiang, Yang Yang, Jican Liu, Wei Hua, Ying Mao, Cheng Li, Weiyue Lu, Jun Qian, Changyou Zhan

**Affiliations:** 1 Department of Pharmacology, School of Basic Medical Sciences & State Key Laboratory of Molecular Engineering of Polymers, Fudan University, 200032 Shanghai, PR China; 2 School of Pharmacy & Key Laboratory of Smart Drug Delivery (Fudan University), Ministry of Education, 201203 Shanghai, PR China; 3 Department of Pathology, Affiliated Zhongshan Hospital Qingpu Branch, Fudan University, 201700 Shanghai, PR China; 4 Department of Neurosurgery, Huashan Hospital Fudan University, 200040 Shanghai, PR China

**Keywords:** Drug delivery, Drug delivery

## Abstract

Protein corona presents a major obstacle to bench-to-bedside translation of targeted drug delivery systems, severely affecting targeting yields and directing unfavorable biodistribution. Corona-mediated targeting provides a new impetus for specific drug delivery by precisely manipulating interaction modes of functional plasma proteins on nano-surface. Here bio-inspired liposomes (SP-sLip) were developed by modifying liposomal surface with a short nontoxic peptide derived from Aβ_1-42_ that specifically interacts with the lipid-binding domain of exchangeable apolipoproteins. SP-sLip absorb plasma apolipoproteins A1, E and J, consequently exposing receptor-binding domain of apolipoproteins to achieve brain-targeted delivery. Doxorubicin loaded SP-sLip (SP-sLip/DOX) show significant enhancement of brain distribution and anti-brain cancer effect in comparison to doxorubicin loaded plain liposomes. SP-sLip preserve functions of the absorbed human plasma ApoE, and the corona-mediated targeting strategy works in SP modified PLGA nanoparticles. The present study may pave a new avenue to facilitate clinical translation of targeted drug delivery systems.

## Abstract (web summary)

Plasma proteins may severely affect the in vivo performance of liposomes. Here, the authors develop bio-inspired liposomes that specifically absorb brain-targeted apolipoproteins and preserve their bioactivities, thereby achieving efficient brain targeting with minor influence on immunocompatibility of liposomes.

## Introduction

During the past several decades, targeted drug delivery systems (TDDS) have gained increasing attention to achieve better therapeutic efficacy and reduced side effects^1–3^. There are numerous examples of TDDS undergoing clinical trials^4^; however, clinical translation of TDDS is relatively slow. Major efforts have so far been made to identify high-affinity ligands^5–7^. After entry into blood stream, the formed protein corona on the surface of TDDS determines what is seen by living organisms, may severely affecting the targeting yields and inducing unfavorable biodistribution^8–12^. Protein corona presents a major obstacle to the bench-to-bedside translation of TDDS.

Besides a large number of inert (or with unknown functions) plasma proteins, there are numerous functional ones absorbing on the surface of drug delivery systems^13,14^. Thus, corona-mediated targeting by preserving the function of target plasma proteins on nano-surface may provide a new impetus for specific drug delivery. Exchangeable apolipoproteins (such as ApoA, C, and E) that can direct the transport of lipids through lymphatic and circulatory systems have been found in protein coronas formed on the surface of a variety of drug delivery systems^15–17^. Some of those exchangeable apolipoproteins (ApoA1 and ApoE) can cross the blood-brain barrier (BBB) via receptor-mediated transcytosis^18–21^; however, it is rarely reported that plain drug delivery systems (without modification of brain-targeting ligands) penetrate the BBB after absorption of such apolipoproteins in blood. One possible reason is that the orientation of functional plasma proteins in the corona layer is random and their accessibility to receptor-binding pocket is impaired^22,23^.

Exchangeable apolipoproteins are typically consisting of two functional domains: lipid-binding domain and receptor-binding domain^24^. We hypothesize that corona-mediated brain targeting would be achievable by precisely manipulating the binding modes of brain-targeting plasma apolipoproteins on nano-surface. Plaques containing β-amyloid (Aβ) peptides are one of the pathological features of Alzheimer’s disease^25,26^, and the clearance of Aβ plaques has been extensively studied during the past decades^27–30^. Several groups have reported that trans-BBB efflux of Aβ into peripheral blood circulation is one of the main pathways of physiological clearance with ApoE, ApoJ, and ApoA1 as chaperones^27,31,32^. The complexes of apolipoproteins and Aβ can traverse the BBB and enter peripheral blood circulation. Short peptide Aβ_25-35_ has been widely studied for its similar effects in Alzheimer disease with its parent peptide Aβ_1-42_^33–35^. Varadarajan et al. have reported that Aβ_25-35_ with amide form of the C-terminal methionine (here termed SP) is devoid of neurotoxicity^36^, providing an ideal candidate for absorption of brain targeting apolipoproteins.

Here, we prepare SP-modified liposomes (SP-sLip) aiming to precisely modulating the composition and functions of the formed protein corona. After entry into blood stream, SP-sLip are anticipated to associate brain targeting apolipoproteins (i.e., ApoE, ApoJ, and ApoA1) via the interaction between SP and the lipid-binding domain of apolipoproteins. Their receptor-binding domains are consequently exposed on the liposomal surface for multiple receptors recognition (LRP1/ApoE, LRP2/ApoJ, and SR-B1/ApoA1) and brain transport via LRP1/LRP2/SR-B1 mediated transcytosis. The brain targeting efficiency and anti-brain cancer effect of doxorubicin-loaded SP-sLip (SP-sLip/DOX) are studied and the application of this novel targeting strategy in other nanomedicines (such as PLGA nanoparticles) is also investigated.

## Results

### SP-sLip rapidly absorb apolipoproteins

**Fig. 1:**
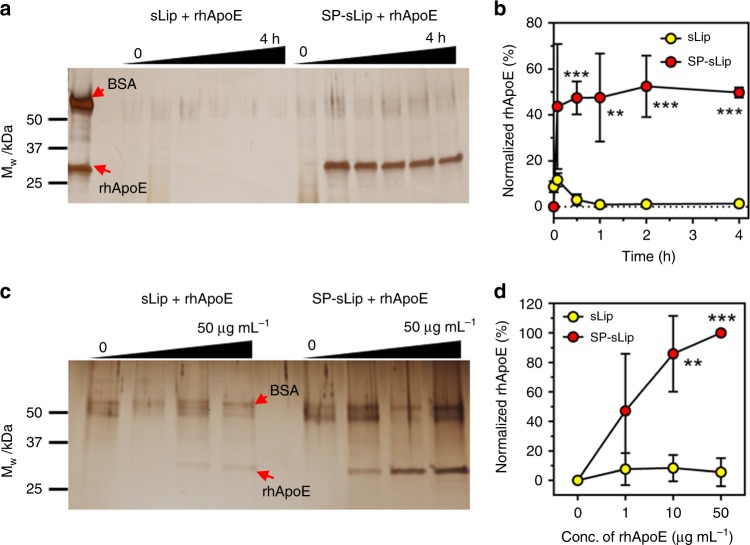
Binding kinetics and capacity of recombinant human ApoE (rhApoE) on liposomal surface. **a**, **b** rhApoE and the same amount of BSA mixture in 100 μL PBS was incubated with 100 μL sLip or SP-sLip for 0, 5, 30, 60, 120, and 240 min at 37 °C (rhApoE, 10 μg mL^−1^; phospholipid, 5 mg mL^−1^). Protein coronas were obtained by centrifugation at 14k RCF for 20 min, loaded to 4–20% SDS-PAGE gel for separation and stained with silver. The band intensity of absorbed BSA and rhAPOE was calculated by image J software and normalized by setting BSA intensity in the control panel as 100. **c**, **d** rhApoE (final concentration of 0, 1, 10, and 50 μg mL^−1^) and the same amount of BSA mixture in 100 μL PBS was incubated with 100 μL sLip or SP-sLip for 1 h at 37 °C. Protein coronas were obtained as in **a**. The band intensity of absorbed rhApoE was calculated and normalized by setting that in the 50 μg mL^−1^ rhApoE panel as 100. Data are means ± SD (*n* = 3), and analyzed with GraphPad Prism 6.0. **p < 0.01, ***p < 0.001 by student’s *t-*test

Thiolated SP (sequence: NH_2_-CGSNKGAIIGLM-CONH_2_) was synthesized and chemically conjugated with mal-PEG3400-DSPE (SP-PEG3400-DSPE, see “Methods”). sLip (5% mol/mol mPEG2000-DSPE, without peptide modification) and SP-sLip (PEGylated liposomes with 2% mol/mol SP-PEG3400-DSPE and 3% mol/mol mPEG2000-DSPE) were prepared using thin-film hydration and extrusion through membranes of 400, 200, and 100 nm pore size^7^. Both liposomal formulations exhibited average sizes of 150 nm. Modification of SP increased the zeta-potential of liposomes from −62.4 to −39.5 mV (Supplementary Table 1), which may be attributed to the positive residue of Lysine and the N-terminal amine group in SP sequence. To study ApoE binding kinetics, sLip and SP-sLip were incubated with recombinant human ApoE (rhApoE, 10 μg mL^−1^). As shown in Fig. 1a, b, SP-sLip could rapidly absorb rhApoE after 5 min incubation at 37 °C, while sLip did not show specific absorption of rhApoE. SP-sLip showed high binding capacity when the concentrations of rhApoE ranging 0–50 μg mL^−1^ (Fig. 1c, d).

**Fig. 2:**
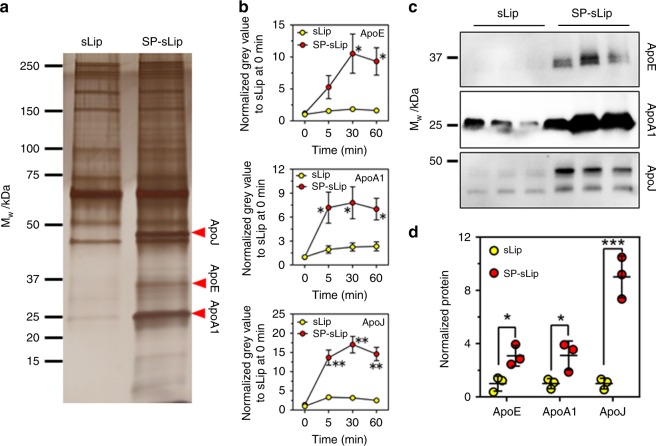
Characterization of protein coronas in vitro and in vivo. **a** sLip or SP-sLip were incubated with mouse plasma for 1 h, and the protein coronas were isolated by centrifugation at 14k RCF, separated by SDS-PAGE and stained with silver. **b** The band intensity of red arrows (ascertained as ApoJ, ApoE, and ApoA1 by nano-LC-MS/MS) was calculated by image J software. The intensity of each apolipoprotein in the formed protein corona on sLip at 0 min was set as 1 for normalization. **c**, **d** In vivo absorption of ApoJ, ApoE, and ApoA1 on liposomal surface. DiI-loaded sLip or SP-sLip (100 μL, DiI 0.4 mg mL^−1^, phospholipid 10 mg mL^−1^) was injected into BALB/c mice via tail vein. Blood was sampled at 1 h after administration and protein coronas were isolated by centrifugation at 14k RCF. Western Blotting was applied to detect the absorbed ApoJ, ApoE, and ApoA1. Quantitative analysis of proteins was performed by Image J software and the average band intensity of each protein in the formed protein corona on sLip was set as 1 for normalization. Data are means ± SD (*n* = 3), and analyzed with GraphPad Prism 6.0. *p < 0.05, **p < 0.01, ***p < 0.001 by student’s *t*-test

The interaction between liposome and mouse plasma was studied. After incubation with mouse plasma for 1 h, the formed protein coronas on the surface of SP-sLip and sLip were carefully collected and plasma proteins were separated using SDS-PAGE (see “Methods”). Three protein bands, which were identified as ApoA1 (30.5 kDa), ApoE (36 kDa), and ApoJ (45 kDa) by nano-LC-MS/MS (Fig. 2a), were found in the protein corona of SP-sLip. To study the binding kinetics of plasma apolipoproteins, SP-sLip were incubated with mouse plasma for different periods and the three apolipoproteins were semi-quantified based on their intensities in SDS-PAGE gels (Fig. 2b). All proteins were specifically absorbed on the surface of SP-sLip with plateaued amount within 5–30 min (30 min for ApoE, 5 min for ApoJ and ApoA1), indicating rapid association of brain-targeting plasma apolipoproteins on SP-sLip. The plasma ApoE binding capacity on liposome surface was studied using ELISA assay (see “Methods”). SP-sLip (2.8 μg ApoE per mg lipid, *p* < 0.05 by student’s *t*-test) displayed significantly higher ApoE binding than sLip (1.6 μg ApoE per mg lipid).

To study whether SP-sLip can absorb apolipoproteins in vivo, BALB/c mice receiving sLip and SP-sLip were killed 1 h after intravenous administration of liposomes. Blood was sampled and the circulating liposomes were separated. The contents of apolipoproteins in the formed protein corona in vivo were quantitated by Western Blotting assay (Fig. 2c, d). For all three apolipoproteins, SP-sLip demonstrated significant increase in comparison to sLip, suggesting that specific absorption of apolipoproteins on SP-sLip occurred in vivo.

### SP-sLip preserve functions of absorbed apolipoproteins

**Fig. 3:**
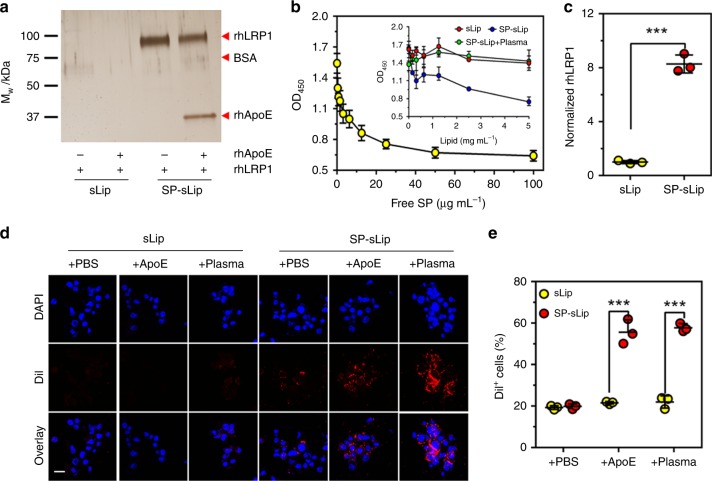
Characterization of ApoE functions in protein corona. **a** sLip or SP-sLip (100 μL, 10 mg mL^−1^ lipid) were incubated with BSA (10 μg mL^−1^) with or without rhApoE (10 μg mL^−1^) for 1 h at 37 °C, then recombinant human LRP1 (rhLRP1) was added and incubated for another 1 h at 37 °C. Protein coronas were isolated by centrifugation at 14k RCF, separated by SDS-PAGE and stained with silver. **b** SP-PEG3400-DSPE dissolved in methanol (0.1 μg per well) was coated in 96-well ELISA plate. After dry in room temperature, it was rinsed by cold PBS for three times. BSA (3%) was used to block for 2 h at room temperature. After thrice PBST rinses, anti-SP antibody (rabbit IgG, containing 0.1% BSA) was added and incubated for 1 h at 37 °C. Different concentrations of free SP (from 0 to 100 μg mL^−1^) or liposomes (sLip or SP-sLip, 0–5 mg mL^−1^ lipid) with or without plasma were incubated for another 1 h at 37 °C. HRP-anti-rabbit IgG was applied to detect the primary antibody using TMB kit. **c** sLip or SP-sLip were incubated with plasma for 1 h at 37 °C, then I^125^ radiolabeled rhLRP1 was added in the mixture for another 1 h incubation. The radioactivity of liposomes after three cycles centrifugation and rinses was counted for detecting the absorbed rhLRP1. **d** Microscopic observation of cellular uptake of DiI-loaded sLip (sLip/DiI) or SP-sLip (SP-sLip/DiI) by bEnd.3 cells. sLip/DiI or SP-sLip/DiI was preincubated with PBS, rhApoE (10 μg mL^−1^) or mouse plasma for 1 h at 37 °C, then incubated with bEnd.3 cells for 4 h at 37 °C. Cellular uptake was detected by confocal laser scanning microscopy. Scale bar = 20 μm. **e** Quantitative analysis of DiI positive bEnd.3 cells by flow cytometry. Data are means ± SD (*n* = 3), and analyzed with GraphPad Prism 6.0. ***p < 0.001 by student’s *t*-test

Aβ peptides have been reported to directly bind LRP1 for clearance^37,38^. The result of SDS-PAGE assay (Fig. 3a) suggested direct interaction between SP-sLip and recombinant human LRP1 (rhLRP1). After entry into blood stream, SP-sLip were immediately surrounded by heavy level of plasma proteins, and SP may be shielded after embedding into the binding pockets of ApoE/ApoJ/ApoA1. In ELISA assay both free SP and SP-sLip could compete the binding of SP-PEG3400-DSPE (immobilized on ELISA wells as antigen) with anti-SP antibody (ab62658, Abcam). After preincubation with mouse plasma, SP-sLip lost this competition activity (Fig. 3b), indicating that plasma proteins reengineered the surface of SP-sLip, where SP was devoid of LRP1 binding activity.

To study whether apolipoproteins on SP-sLip surface preserve bioactivity or not, SP-sLip, and sLip were preincubated with the equal volume of fresh mouse plasma for 1 h, followed by incubation with I^125^ radiolabeled rhLRP1 (see “Methods”) for 1 h at 37 °C. As shown in Fig. 3c, SP-sLip could absorb 8.4-fold rhLRP1 of sLip after preincubation with the equal volume of mouse plasma, suggestive of preservation of bioactivity of absorbed ApoE. To evaluate the targeting capacity, DiI-labeled SP-sLip and sLip pretreated with phosphate-buffered saline (PBS) only, rhApoE in PBS (10 μg mL^−1^), or 50% of mouse plasma were incubated with mouse brain capillary endothelial cells (bEnd.3) for 4 h in DMEM. As shown in Fig. 3d, e, sLip in PBS did not show significant endocytosis by bEnd.3 cells. Neither the addition of rhApoE nor mouse plasma-induced enhanced cellular uptake of sLip. In contrast, SP-sLip demonstrated low uptake by bEnd.3 cells with the pretreatment of PBS only, suggesting that direct interaction between SP and LRP1 may be weak to induce efficient cellular uptake. The addition of rhApoE or mouse plasma significantly boosted endocytosis of SP-sLip by bEnd.3 cells. The present results indicate that SP-sLip are capable of preserving the activity of absorbed apolipoproteins for brain targeting.

### SP-sLip can penetrate BBB and target brain cancers

**Fig. 4:**
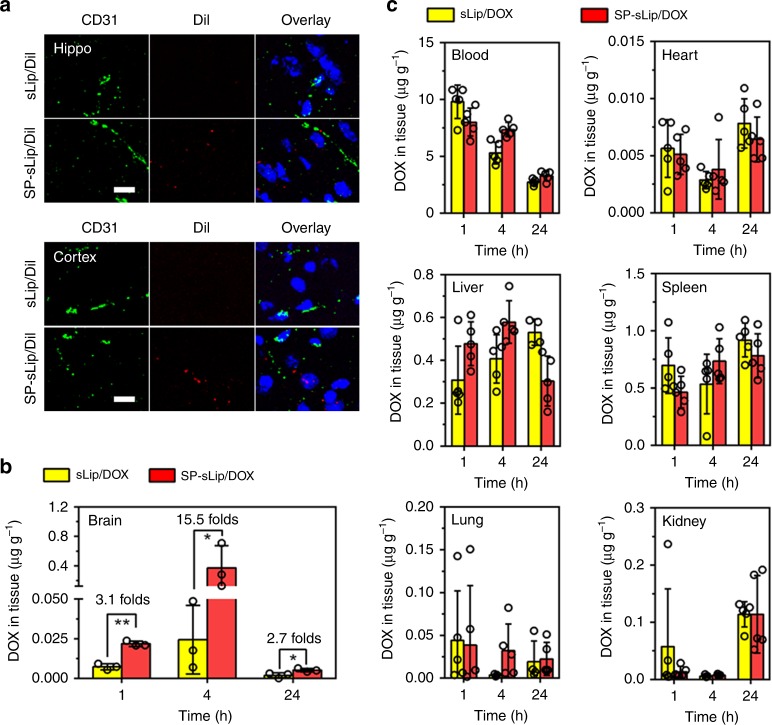
Biodistribution of sLip and SP-sLip in healthy BALB/c mice. **a** DiI-labeled sLip (sLip/DiI) and SP-sLip (SP-sLip/DiI) were injected via tail vein and brains were dissected and sectioned at 4 h after administration. Brain slices were stained with anti-CD31 antibody (green) and biodistribution of liposomes (red) in hippocampus (Hippo) and cortex were observed by confocal laser scanning microscopy. Scale bar = 20 μm. Doxorubicin distribution in brain (**b**) and other main organs (**c**) at 1 h, 4 h, and 24 h after intravenous injection of SP-sLip/DOX and sLip/DOX. Data are means ± SD (*n* = 3–5). *p < 0.05, **p < 0.01 by student’s *t*-test

The brain targeting capability of SP-sLip was studied in healthy BALB/c mice. DiI-labeled SP-sLip and sLip were intravenously injected through the tail vein and mice were killed 4 h after administration. Brains were dissected and frozen sectioned. SP-sLip showed significant distribution in hippocampus and cortex (Fig. 4a) in comparison to sLip. To further quantify the brain targeting efficiency and biodistribution of SP-sLip, doxorubicin-loaded SP-sLip (SP-sLip/DOX), and sLip (sLip/DOX) were intravenously injected into the tail vein of healthy BALB/c mice and the main organs were dissected at 1, 4, and 24 h after injection. As shown in Fig. 4b, SP-sLip/DOX displayed significantly higher brain distribution of doxorubicin at all tested time points than sLip/DOX. In particular, doxorubicin exhibited the peak brain distribution in both groups 4 h after injection. SP-sLip/DOX (ID% g^−1^ = 0.93%) treated mice had 14.5-fold higher brain doxorubicin distribution than sLip/Dox (ID% g^−1^ = 0.061%) treated mice. SP-sLip/DOX and sLip/DOX displayed comparable biodistribution of doxorubicin in other main organs (Fig. 4c). Biosafety of doxorubicin-loaded liposomes after intravenous injection was evaluated after five injections of SP-sLip/DOX and sLip/DOX (doxorubicin 2 mg kg^−1^, intravenous injections every 2 days). All the main organs were dissected and subject to hematoxylin and eosin (H&E) staining (Supplementary Fig. 1). Neither SP-sLip/DOX nor sLip/DOX induced perceptible toxicity. Liver functions of these mice after 10 days treatment of DOX-loaded liposomes were analyzed using liver functions detection kits (Supplementary Fig. 2), indicating that SP-sLip/DOX and sLip/DOX did not cause any liver disorders.

**Fig. 5:**
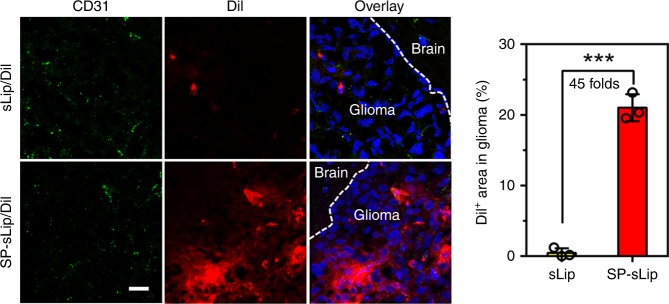
Biodistribution of sLip and SP-sLip in intracranial glioma. DiI-labeled sLip (sLip/DiI) and SP-sLip (SP-sLip/DiI) were injected via tail vein of nude mice bearing intracranial glioma (at day 16 after implantation). Mouse brains were dissected and sectioned at 4 h after administration. Brain slices were stained with anti-CD31 antibody (green) and biodistribution of liposomes (red) in normal brain tissues (labeled with Brain) and glioma region (labeled with Glioma) were observed by confocal laser scanning microscopy and quantified by Image Pro. Scale bar = 20 μm. Data are means ± SD (*n* = 3). ***p < 0.001 by student’s *t*-test

**Fig. 6:**
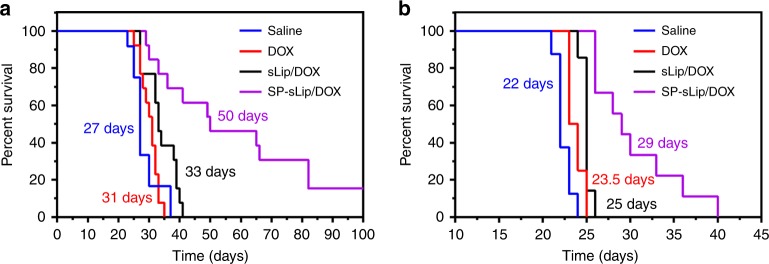
Kaplan–Meier survival curve of nude mice bearing intracranial brain cancer cells after treatments. For mouse bearing U87 cells (**a**), saline (27 days, *n* = 12), DOX (31 days, *n* = 13), sLip/DOX (33 days, *n* = 13), and SP-sLip/DOX (50 days, *n* = 13) were intravenously administered at a total DOX dose of 10 mg kg^−1^ (injections at day 7, 9, 11, 13, and 15 after U87 cells implantation). For mouse bearing D425 (**b**), saline (22 days, *n* = 8), DOX (23.5 days, *n* = 8), sLip/DOX (25 days, *n* = 7), and SP-sLip/DOX (29 days, *n* = 9) were intravenously administered at a total DOX dose of 12 mg kg^−1^ (injections at day 3, 5, 7, 10, 12, and 14 days after D425 cells implantation). Data were plotted and the median survival time was calculated using GraphPad Prism 6.0 (Log-rank (Mantel–Cox) test)

Glioma targeting capacity of SP-sLip was evaluated in a xenograft nude mouse model of human glioblastoma multiforme (GBM, see “Methods”). DiI-labeled sLip demonstrated scattered distribution in the glioma region (Fig. 5a, b). In sharp contrast, SP-sLip showed a 45-fold increase of distribution than did sLip. These results suggest that SP-sLip are capable of penetrating in vivo BBB and targeting intracranial glioma. Besides on the BBB, LRP1 was also found to be highly expressed on glioma cells^39–41^. Intracranial glioma accumulation of SP-sLip may be at least partially attributed to LRP1 mediation. To evaluate the potential therapeutic value of SP-sLip for glioma, we studied the therapeutic efficacy of liposomal formulations encapsulating doxorubicin in nude mice bearing intracranial human glioma cells (U87). Four groups of nude mice (*n* = 12–13) bearing intracranial U87 cells were intravenously injected with saline, free DOX, sLip/DOX, and SP-sLip/DOX at day 7, 9, 11, 13, and 15 after tumor implantation. As shown in Fig. 6a, in the absence of SP, treatments with free or liposome-formulated DOX at a total dose of 10 mg per kg body weight did little in improving mouse survival, registering a median survival of 31 days (*p* = 0.1563 by Log-rank (Mantel–Cox) test) and 33 days (*p* = 0.005 by Log-rank (Mantel–Cox) test) versus 27 days of the saline treated group. SP-sLip/DOX significantly prolonged the median survival time of nude mice to 50 days (*p* < 0.0001 vs. saline treatment by Log-rank (Mantel–Cox) test). In comparison to sLip/DOX, SP-sLip/DOX lengthened the survival of model mice by extending additional 17 days (283% of the prolongation resulted from the treatment of sLip/DOX, *p* < 0.01 by Log-rank (Mantel–Cox) test) of median survival time of intracranial U87-bearing nude mice, in which BBB is the main obstacle to efficient drug delivery. Two out of the thirteen model mice receiving SP-sLip/DOX kept healthy at the termination of survival experiments (100 days after tumor implantation). Both brains were dissected, sectioned, and stained with H&E. As shown in Supplementary Fig. 3, no tumor could be observed in brains, indicating that SP-sLip/DOX cured glioma of those two model mice. All these results suggest high potency of SP-sLip/DOX for glioma targeted chemotherapy.

To understand the anti-glioma mechanism, mice receiving various DOX formulations were killed at 16 days post tumor implantation and the brains were dissected for anti-CD31 antibody and TUNEL staining. As shown in Supplementary Fig. 4, the results clearly showed that SP-sLip/DOX could ablate glioma blood vessels and induce apoptosis of glioma cells. Body weight of nude mice after receiving different DOX formulations was monitored, and the results shown in Supplementary Fig. 5 indicated none of those formulations induced significant decrease of mice body weight in comparison to saline-treated group.

The targeting capability of SP-sLip/DiI was also evaluated in a xenograft nude mouse model bearing human medulloblastoma cells. D425 is a human patient-derived medulloblastoma cell line with high malignancy^42,43^. As shown in Supplementary Fig. 6, SP-sLip/DiI exhibited significant accumulation in the D425 (GFP labeled) tumor region. SP-sLip/DiI also demonstrated higher distribution in nontumor region of the brain than sLip/DiI, particularly around the brain blood vessels. To evaluate the therapeutic value of SP-sLip/DOX for medulloblastoma, four groups of nude mice (*n* = 7–9) bearing intracranial D425 cells were intravenously injected with saline, free DOX, sLip/DOX, and SP-sLip/DOX at day 3, 5, 7, 10, 12, and 14 after tumor implantation (see “Methods”). As shown in Fig. 6b, treatments with free DOX or sLip/DOX at a total dose of 12 mg per kg body weight slightly improved mouse survival, registering a median survival of 23.5 days (*p* = 0.0129 by Log-rank (Mantel–Cox) test) and 25 days (*p* = 0.0002 by Log-rank (Mantel–Cox) test) versus 22 days of the saline treated group. SP-sLip/DOX significantly extended the median survival time of nude mice to 29 days (*p* < 0.0001 vs. saline treatment by Log-rank (Mantel–Cox) test). In comparison to sLip/DOX, SP-sLip/DOX lengthened the survival of model mice by prolonging additional 4 days (133% of the prolongation resulted from the treatment of sLip/DOX, p < 0.001 by Log-rank (Mantel–Cox) test) of median survival time of intracranial D425-bearing nude mice.

### SP-sLip are immunocompatible

**Fig. 7:**
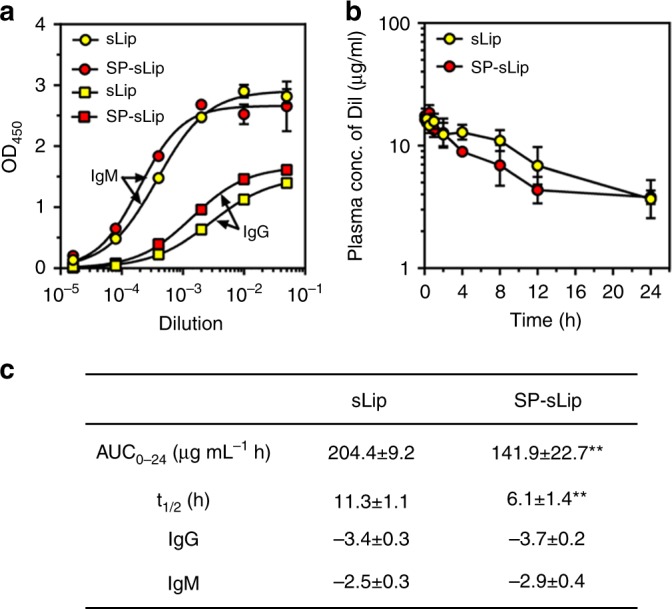
Evaluation of immunocompatibility of SP-sLip. **a** Immune response caused by sLip or SP-sLip in C57BL/6 mice. Absorbance in the ELISA plate versus serum dilution and antibody titer reported as log (EC50). mPEG2000-DSPE and SP-PEG3400-DSPE were used as antigens for sLip and SP-sLip, respectively. Data are means ± SD (*n* = 5). **b** Pharmacokinetic profile of DiI-labeled sLip and SP-sLip in SD rats over 24 h after intravenous injection. Data are means ± SD (*n* = 4) **c** PK parameters are calculated by DAS 2.0, and titration of IgG and IgM was calculated using GraphPad Prism 6.0. **p < 0.01 by student’s *t*-test

SP is the amide form of Aβ_25–35_, which was previously reported to be nontoxic to neural cells^36^. Here we also confirmed that SP was nontoxic to PC12 cells after 48 h incubation even at the concentration of 100 μg mL^−1^ (Supplementary Fig. 7). The immunogenicity of SP-sLip was studied in C57BL/6 mice by detecting the titration of IgG and IgM (see “Methods”). After four doses, SP-sLip, and sLip induced comparable titration levels of IgG and IgM (Fig. 7a, c), suggesting that SP modification did not generate enhanced immunogenicity. In addition, intravenous injection of liposomes (50 mg kg^−1^) into BALB/c mice did not induce enhancement of plasma IL6 level (Supplementary Fig. 8), indicating no acute proinflammatory cytokine response after liposomes administration.

The pharmacokinetic (PK) profile of SP-sLip was studied. As shown in Fig. 7b, c, SP-sLip demonstrated relatively rapid clearance in Sprague–Dawley rats than sLip, registering 30% decrease of the area under the curve (AUC_0–24_) and 46% decrease of circulation half-life (*t*_1/2_). Apolipoproteins have been reported as dysopsonins^44,45^. Absorption of apolipoproteins would be favorable for enhanced immunocompatibility of nanocarriers. The change of PK profile of SP-sLip may relate to its favorable distribution in brain, as well as slight enhancement of distribution in liver (Fig. 4).

### SP modification presents a novel targeting platform

**Fig. 8:**
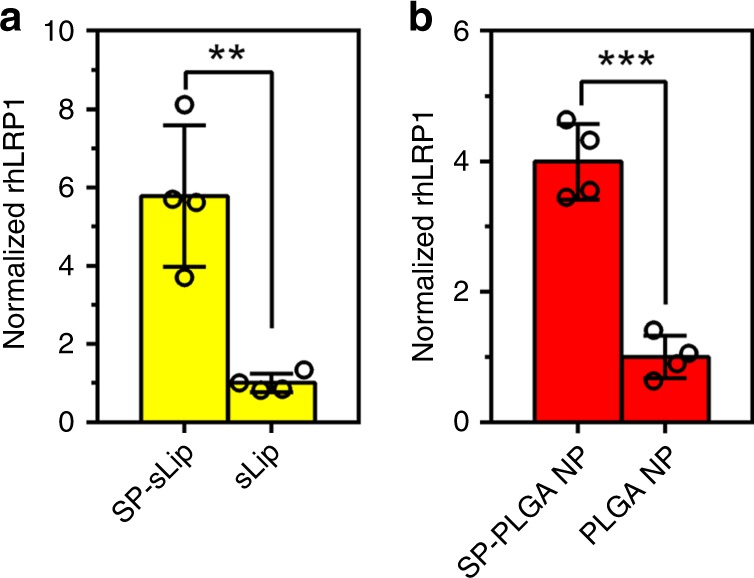
Characterization of ApoE function in protein corona formed in human plasma. sLip and SP-sLip (**a**), or plain PLGA nanoparticles (PLGA NP) and SP-modified PLGA nanoparticles (SP-PLGA NP) (**b**) were incubated with human plasma for 1 h at 37 °C, then I^125^ radiolabeled rhLRP1 was added in the mixture for another 1 h incubation. The radioactivity of liposomes and PLGA nanoparticles after three cycles centrifugation and rinses was counted for detecting the absorbed rhLRP1. Data are means ± SD (*n* = 4), **p < 0.01, ***p < 0.001 by student’s *t*-test

In the aforementioned experiments, SP-sLip have been verified to absorb rhApoE, preserving its rhLRP1-binding activity. We further studied whether SP-sLip could absorb ApoE in human blood and preserve its binding affinity with rhLRP1. SP-sLip and sLip were incubated with healthy human plasma for 1 h, and further incubated with I^125^ radiolabeled rhLRP1 (see “Methods”) for another 1 h. The absorbed radiolabeled rhLRP1 on liposomal surface was separated by centrifugation and determined using a gamma counter. As shown in Fig. 8a, SP-sLip could efficiently interact with rhLRP1 after incubation with human plasma, indicating that absorbed ApoE from human plasma preserves receptor-binding activity. SP was also modified on the surface of PLGA nanoparticles (SP-PLGA NP, see “Methods” and Supplementary Table 1), which are also widely used as nanocarriers for chemotherapeutics^46,47^. As expected, SP-PLGA NP could interact with rhLRP1 after incubation with human plasma (Fig. 8b). These results suggest that SP modification can serve as a platform for design of TDDS.

## Discussion

Liposome-based TDDS have been widely investigated in preclinical and clinical stages. Thorough studies on the formation of protein corona on liposomal surface and consequential effects on targeting yielding and immunocompatibility would be crucial to facilitate the bench-to-bedside translation. PEGylation is among the most popular methods for the preparation of antifouling nano-surface, thus yielding long-circulating liposomes. Unfortunately, plasma protein absorption on liposomal surface is inevitable even in the presence of PEG^8,48,49^. Modification of targeting ligands on the distal end of PEG is widely used to functionalize liposomal surface, which would severely affect the formation of protein corona (including protein type, concentration, and conformation). Recently we found the modification of long, stable positively charged peptide ligand on PEGylated liposomes induced enhanced absorption of IgM and unfavorable immunocompatibility^50,51^.

Brain-targeting plasma proteins, such as transferrin and ApoE, have been used as targeting ligand for brain-targeted drug delivery^52–55^. However, such researches are restricted in preclinical stage. Direct modification of brain targeting plasma proteins on the surface of nanocarriers may encounter the following drawbacks for clinical translation^56–58^: (1) Protein modification make it costly in production, storage, and transportation; (2) Protein modification is risky to elevate immunogenicity; (3) Endogenous brain targeting plasma protein competes receptor binding of TDDS; (4) The last but not the least, protein corona formed on modified nanomedicines may further affect targeting yield.

In the present study, corona-mediated brain-targeting has been achieved using a short peptide for association of exchangeable apolipoproteins in an established pattern. Receptor binding domains of apolipoproteins are appropriately exposed on liposomal surface after entry of liposomes into blood stream. The reengineered liposomes in vivo demonstrate high brain-targeting capacity and efficiently facilitate brain cancer-targeted therapy. This strategy demonstrates efficiency in human plasma, and SP modification on polymeric nanoparticles can also reengineer the nano-surface for brain-targeting property. This proof-of-concept implementation suggests that target plasma proteins are theoretically possible to be utilized to reengineer the nano-surface of drug delivery systems.

## Methods

### Animals and cells

Sprague–Dawley rats, C57BL/6 mice, BALB/c, and nude mice were purchased from Shanghai SLAC laboratory animal Co. LTD (Shanghai, China) and kept under SPF conditions. All animal experiments were carried out in accordance with guidelines evaluated and approved by the ethics committee of Fudan University. PC12 cells, bEnd.3 cells, and U87 cells were obtained from Shanghai Institute of Cell Biology. D425 cell line was provided by Prof. Yujie Tang at Shanghai Jiaotong University. Cells are maintained in Dulbecco’s Modified Eagle Medium (Gibco) supplemented with 10% FBS (Gibco), 100 U mL^−1^ penicillin, and 100 μg mL^−1^ streptomycin at 37 °C under a humidified atmosphere containing 5% CO_2_.

### Compounds and antibodies

HSPC (hydrogenated soy phosphatidylcholine), mPEG2000-DSPE, and cholesterol were purchased from A.V.T. (Shanghai) Pharmaceutical Co., Ltd. Fmoc-protected amino acids for solid-phase peptide synthesis were from CSBio (Shanghai) Ltd. Mal-PEG3400-DSPE was obtained from Laysan Bio Co. (Arab, AL). Sephadex G50 and DiI (DiIC18(3), a fluorescent lipophilic cationic indocarbocyanine dye) was purchased from Sigma (St. Louis, MO). DAPI, TUNEL kit, TMB Horseradish Peroxidase Color Development Solution for ELISA and Fast Silver Stain Kit was from Beyotime Biotechnology (Nantong, China). Doxorubicin hydrochloride was purchased from Zhejiang Haizheng Co. (Zhejiang, China). Recombinant human ApoE was from PEPROTECH (Rocky Hill, NJ). Recombinant human LRP1 Cluster II Fc Chinera, Human IgG1 was from R&D system (Minneapolis, MN). Recombinant human LRP1 antibody rabbit polyclone IgG, apolipoprotein E antibody rabbit polyclone IgG (ab92544), anti-clusterin (ApoJ) goat polyclone IgG (ab130386), anti-Apolipoprotein A1 antibody rabbit polyclone IgG (ab20453), CD31 antibody rabbit polyclone IgG (ab28364), anti-beta Amyloid antibody rabbit polyclone antibody (ab62658), HRP-rabbit-anti-goat IgG (H&L, ab6741), Alexa Fluor 488-Goat anti-Rabbit IgG H&L (ab150077), mouse ApoE ELISA kit (ab215086), and Alexa Fluor 405-Donkey anti-rabbit IgG H&L (ab175651) were purchased from Abcam (Cambridge, UK). Anti-laminin rabbit IgG was acquired from Sigma-Aldrich (St.Louis, MO). ApoE polyclonal antibody Goat/IgG for human species, mouse IL-6 uncoated ELISA kit (88–7064) were from Thermo Fisher (Waltham, MA). Peroxidase-conjugated AffiniPure Goat anti-Rabbit IgG(H + L) (ZB-2301) and peroxidase-conjugated AffiniPure Goat anti-mouse IgG(H + L) (ZB-2305) were from ZSGB-BIO (Beijing, China). Aspartate aminotransferase assay kit(C010–1), Alanine aminotransferase assay kit(C009–1), Alkaline phosphatase assay kit(A059–2), Urea assay kit(C013–1), and Creatinine assay kit(C011–1) were from Nanjing Jiancheng Bioengineering Institute (Nanjing, China).

### Synthesis of SP-PEG3400-DSPE

SP peptide with an additional cysteine in the N-termini was synthesized using a solid phase peptide synthesizer (CS336, CSBio (Shanghai) Ltd.) and purified by preparative HPLC^59^. To synthesize SP-PEG3400-DSPE, mal-PEG3400-DSPE (50 mg) was dissolved in 5 mL chloroform and rotary evaporated to form a thin film. Distilled water (DW, 3 mL) was added to hydrate the film for 1 h at 37 °C. Thiolated SP (26 mg) was dissolved in 2 mL DW, and mixed with mal-PEG3400-DSPE suspension, then 3 mL 0.1 M phosphate-buffered solution containing 40 μL EDTA (500 mM, pH 8.0) was added in the mixture, followed by 6 h incubation at room temperature. The residual peptide and salt were removed by dialysis against DW (MWCO 8000 Da) for 72 h. SP-PEG3400-DSPE was obtained by freeze-drying.

### Preparation of sLip and SP-sLip

Liposomes were prepared by the thin-film hydration and extrusion method. For blank liposomes (without dye or drug loading), a mixture of HSPC/cholesterol/mPEG2000-DSPE (52/43/5 in molar ratio) or HSPC/cholesterol/mPEG2000-DSPE/SP-PEG3400-DSPE (52/43/3/2 in molar ratio) in CHCl_3_ was rotary evaporated to form a thin film. Any residual organic solvent was removed by overnight evaporation under vacuum. The dried lipid film was subsequently hydrated with saline at 60 °C for 1 h, and the lipid dispersion was extruded through polycarbonate membranes with the pore size ranging from 400 to 100 nm. DOX-loaded liposomes were prepared using a traditional ammonium sulfate gradient loading method^60,61^. Briefly, the dried lipid film was hydrated with 0.32 M ammonia sulfate at 60 °C for 1 h, and the lipid solution was extruded through polycarbonate membranes with pore size of 400, 200, and 100 nm. The external liquid phase was replaced with saline using G50 column, then the liposomes were incubated with doxorubicin solution at the ratio of 10 to 1 (lipid/DOX, w/w) at 60 °C for 15 min. After cool down to room temperature, free doxorubicin was removed by G50 column.

### Preparation of PLGA nanoparticles

PLGA (16 mg), mPEG-DSPE (4 mg), and mPEG-PLGA (4 mg) were dissolved in 0.5 mL dichloromethane and added into 2 ml 0.5% sodium cholate under sonication for 5 min in an ice–water bath to form oil-in-water emulsion. Dichloromethane was evaporated by rotary evaporation. The prepared PLGA nanoparticles were collected by centrifugation at 10k RCF for 25 min. SP-modified nanoparticles were prepared using the similar method except for the addition of SP-PEG3400-DSPE (1.2 mg).

### Binding of ApoE on SP-sLip in vitro

Recombinant human ApoE was mixed with equal amount of BSA in PBS, and incubated with sLip or SP-sLip (final concentration 5 mg mL^−1^ phospholipid) with different periods and rhApoE concentrations at 37 °C. The sample was centrifuged at 14k RCF for 20 min to pellet the liposome-protein complexes, which was washed with 300 μL cold PBS and transferred into a new Protein LoBind tube. The wash procedure was repeated thrice.

Proteins were desorbed from liposomes by adding SDS-PAGE sample buffer (5 × SB, Beyotime) to the pellet and boiled at 95 °C for 10 min. A 4–20% polyacrylamide gel (Biorad) was used to separate proteins. Electrophoresis was carried out at a constant current (20 mA gel^−1^) in electrophoresis buffer, until the dye front reached the lower end of the gel (1 h). Proteins were fixed on gel with H_2_O/CH_3_OH/CH_3_COOH (50:45:5, v/v/v) for 1 h under gentle agitation. Ag staining-kit (Beyotime) was used to stain gels under agitation according to the manufacturer’s manual (see Supplementary Figs. 9 and 10).

### Isolation of protein corona in vitro and in vivo

Samples of mouse whole blood were obtained from BALB/c mice with Na_2_EDTA anticoagulant and protease inhibitors cocktail (Roche). Mouse plasma was prepared by centrifugation at 1000 × g for 10 min, pooled and split into 100 μL aliquots, and stored at −80 °C in labeled Protein LoBind tubes until further use. For analysis, the aliquots were thawed at 4 °C and then allowed to warm at room temperature.

Plasma protein binding to liposomes was studied by incubating 100 μL liposome suspension (10 mg mL^−1^) in PBS with 100 μL plasma at 37 °C for 1 h. The protein corona was isolated by centrifugation at 14k RCF and three washes with cold PBS, then separated by SDS-PAGE, stained with silver kit as aforementioned in ApoE absorption experiment (see Supplementary Fig. 11). All the experiments were triplicated independently to verify the reproducibility of the liposome-protein complex pellet size, general pattern, and band intensity.

### In-gel digestion and nano-LC-MS/MS identification

The entire gel was cut into pieces at the target sites (as red arrows shown in Fig. 2a), which were destined, reduced, and alkylated, followed by trypsin digestion. The digested peptides were extracted, resuspended in 0.1% formic acid, and analyzed by nano-LC-MS/MS on an LTQ Orbitrap Fusion mass spectrometer (Thermo Electron, San Jose, CA). The raw data were processed using Proteome Discoverer (version 1.4, Thermo Fisher Scientific Inc.) and matched with the UniProt mouse protein database (version 20130507) using the Mascot search engine (version 2.3.01, Matrix Science, London, UK). The fixed modification was set to cysteine alkylation. The N-terminal acetylation and methionine oxidation of plasma protein were set as variable modifications. The minimal scores for peptide and protein were set as 20 and 50, respectively. The false discovery rate was set to 0.01 for analysis.

### In vivo analysis of protein corona of liposomes

DiI-labeled sLip and SP-sLip (DiI 0.4 mg mL^−1^, phospholipid 10 mg mL^−1^) were injected via the tail vein of BALB/c mice. Whole blood was sampled with EDTA anticoagulant in Protein LoBind tubes at 1 h after administration, and plasma was separated by centrifugation at 1000 × *g* for 10 min. Liposomes were normalized by detecting the fluorescence intensity of DiI (Ex 555 nm, Em 575 nm). The isolated protein coronas absorbed on liposomes (procedure was the same as in vitro) were subjected to Western Blotting analysis. Briefly, after separation by 4–20% SDS-PAGE, the samples were transferred to PDVF membrane, and blocked by 5% nonfat milk in PBST (PBS containing 0.1% Tween-20) for 1 h at room temperature. Primary antibodies (1:1000 for anti-ApoJ, 1:2000 for anti-ApoE, 1:5000 for anti-ApoA1) were incubated with the membrane overnight at 4 °C. Following by three times wash with PBST, the membranes were incubated with horseradish conjugated secondary antibodies (1:2000 anti-goat IgG for ApoJ, 1:5000 anti-rabbit IgG for ApoE and ApoA1) at room temperature for 1 h. After six washes of PBST, enhanced ECL kit (Millipore) was used to react with the membrane, and the signal was imaged (ChemiScope 6000, Clinx Co. Ltd) (see Supplementary Figs. 12–14). The data were analyzed by Image J software.

### Assessment of activities of SP-sLip and absorbed ApoE

Liposomes (100 μL, 10 mg mL^−1^ of phospholipid) were preincubated with or without rhApoE at 37 °C for 1 h, then rhLRP1 (1 μg) was added for another 1 h incubation at 37 °C. The pellets were collected by centrifugation at 14k RCF for 20 min, and rinsed with 300 μL cold PBS thrice. Pellet was resuspended in 30 μL PBS, and mixed with sample buffer containing β-Mercaptoethanol. The SDS-PAGE and silver staining were conducted as aforementioned (see Supplementary Fig. 15).

In ELISA assay, SP-PEG3400-DSPE in methanol was immobilized in 96-well ELISA plates (0.1 μg per well). After volatilization, cold PBS was added to wash for three times. All wells were blocked with 3% BSA in PBS for 2 h at room temperature, followed with thrice PBST rinses. Anti-SP antibody (ab62658, 1:1000) in 0.1% BSA was added and incubated for 1 h at 37 °C. sLip or SP-sLip (10 mg mL^−1^ phospholipid) were preincubated with or without mouse plasma (1:1 v/v) for 1 h at 37 °C, then diluted to different concentrations ranging 0–5 mg mL^−1^ and added to the wells. Free SP with concentrations from 0 to 100 μg mL^−1^ was added as control. After washes, HRP-anti-rabbit IgG (ZB-2301, 1:5000) was used to detect the primary antibody using TMB kit.

ELISA assay was conducted to quantify the absorbed ApoE on SP-sLip and sLip. In brief, liposomes (100 μL, 7.5 mg mL^−1^ of phospholipid) were incubated with mouse plasma (50 μL) at 37 °C for 1 h. After centrifugation at 14k RCF for 20 min, the supernatant was collected for detecting the remaining ApoE in plasma using ApoE ELISA kit according to the manufacturer’s manual.

Liposomes (100 μL, 10 mg mL^−1^ of phospholipid) or PLGA nanoparticles (100 μL, 10 mg mL^−1^ of PLGA) were incubated with plasma (100 μL, of mouse or human) at 37 °C for 1 h, and I^125^ radiolabeled rhLRP1 (6 μCi, 0.5 μg protein) was added for another 1 h incubation at 37 °C. The sample was centrifuged at 19k RCF for 30 min to pellet the protein corona complexes, which were washed with 300 μL cold PBS and transferred into a new Protein LoBind tube. The wash procedure was repeated three times. Pellet was suspended in 500 μL PBS, and the radioactivity was measured using a gamma counter.

### Cellular Uptake

bEnd.3 cells (1 × 10^6^ per well) were seeded onto a six-well plate. After 2 days of cultivation, cells were subsequently incubated with DiI-labeled sLip or SP-sLip (0.2 mg mL^−1^ phospholipid) (liposomes were preincubated with PBS, 10 μg mL^−1^ rhApoE, or equal volume of mouse plasma at 37 °C for 1 h) at 37 °C for 4 h in culture medium without FBS. After three rinses with PBS, the fluorescence intensity was captured by a LSM710 laser scanning confocal microscope (ZEISS) and quantified by a FACS Aria II flow cytometer (BD Biosciences).

### Intracranial U87-bearing mouse model

The orthotropic glioma model was established by implantation of U87 cells in brains of nude mice^62^. Briefly, male nude mice (18–20 g) were anesthetized with chloral hydrate and U87 cells (8 × 10^5^ cells suspended in 5 μL PBS) were implanted into the right brain (0.6-mm anterior, 1.8-mm lateral to the bregma with 3-mm depth) using a stereotactic apparatus (Stoteling). The injection speed was 1 μL per min.

### Intracranial D425-bearing mouse model

D425 cells were digested to single cell with TRPLE (Gibco) and DNase I (Roche), and implanted into right cerebellum of nude mice (2 × 10^4^ cells suspended in 3 μL PBS). The stereotactic position coordinated 1 mm to the midline, 2 mm posterior to the lambdoid, and 2.15 mm in depth.

### Biodistribution of liposomes in vivo

DiI-labeled sLip and SP-sLip were injected into healthy BALB/c mice and tumor-bearing nude mice (16 days after tumor implantation of U87-bearing model and 18 days after implantation of D425-bearing model) via the tail vein. After 4 h, brains were harvested, dehydrated, and subsequently frozen in Tissue-Tek^®^ O.C.T. compound. Frozen sections of 15 μm thickness were prepared and ruptured with 0.5% Triton X-100 for 15 min. For the U87-bearing mouse model, microvessels were labeled with anti-CD31 antibody (ab28364, 1:50) overnight at 4 °C after blockage with 3% BSA for 1 h, and stained with an Alexa Fluor 488-conjugated secondary antibody (ab150077, 1:250) for 1 h at 37 °C and 300 nM DAPI for 10 min at room temperature. For D425-bearing mouse model, microvessels were labeled with anti-laminin antibody (sigma, L9393, 1:25) overnight at 4 °C and detected with Alexa Fluro 405-conjugated secondary antibody (ab175651, 1:250) for 1 h at room temperature. The sections were examined under a DMI4000D fluorescence microscope (Leica). Image Pro software was used to quantify brain distribution of liposomes.

SP-sLip/DOX and sLip/DOX were intravenously injected into BALB/c mice at a dose of 2 mg kg^−1^. After 1 h, 4 h, and 24 h, blood was sampled and mice were perfused using cold PBS to remove blood from organs. The brain, heart, liver, lung, and kidney were dissected and stored in −80 °C until use. The tissues were weighed, homogenized with distilled water at 4 °C, and doxorubicin was extracted using 500 μL CHCl_3_, 100 μL methanol and 50 μL inner standard (10 μg mL^−1^ daunomycin in methanol). After vortex and centrifugation, the CHCl_3_ phase was collected and transferred into a new tube. Chloroform was air-dried and the samples were suspended in methanol. After centrifugation the supernatant was injected to HPLC doxorubicin detection.

### Pharmacodynamics study

Nude mice bearing U87 cells were divided into four groups randomly (*n* = 12–13). From the 7th day after tumor implanted, saline, free DOX, sLip/DOX, or SP-sLip/DOX (DOX 2 mg kg^−1^) was injected from tail vein for five times (at 7, 9, 11, 13, and 15 days post tumor implantation). Body weight and survival time were recorded. Median survival time was calculated with GraphPad Prism 6.0.

At day 16, three mice per group were anesthetized and perfused with cold PBS. Brains were dissected, fixed with 4% PFA and 30% sucrose. The samples were embedded in Tissue-Tek^®^ O.C.T. compound and frozen sectioned in 15-μm thickness. TUNEL kit was applied to stain the cell apoptosis in tumor area. Sections were washed with cold PBS for three times, and blocked with 3% BSA in PBS for 1 h in room temperature and ruptured with 0.5% Triton X-100 for 15 min. CD31 antibody (ab28364, 1:50 dilution in 0.1% BSA) was incubated with the brains overnight at 4 °C, then Alex-488 goat-anti-rabbit IgG (ab150077, 1:250) was applied to detect the primary antibody after PBS washes. DAPI was used to stain the brain slices for 10 min at room temperature. The signal was visualized in a DMI4000D fluorescence microscope (Leica). Image Pro software was used to quantify the apoptosis area and microvessels in tumor.

Nude mice bearing D425 cells were divided into four groups. From the 3rd day after tumor implantation, the saline, free DOX, sLip/DOX or SP-sLip/DOX (DOX 2 mg kg^−1^), was intravenously administered for six times (at 3, 5, 7, 10, 12, and 14 days after implantation). The survival and body weight of mice were monitored and the median survival time was calculated with GraphPad Prism 6.0.

### Pharmacokinetics study

To evaluate the circulation half-life of sLip and SP-sLip, DiI-labeled liposomes were injected into SD rats via the tail vein (*n* = 4). Blood was sampled at 0, 5, 15, 30 min, and 1, 2, 4, 8, 24 h after injection, and centrifuged at 1000 × g for 10 min to collect plasma. The plasma samples were diluted with normal plasma in a 96-well plate for fluorescence measurements using a Synergy 2 microplate reader (Bio-tek). Pharmacokinetic parameters were calculated using the DAS 2.0 software.

### Evaluation of immunogenicity

Lipid A loaded sLip or SP-sLip were intraperitoneally injected to C57BL/6 mice every 7 days for four times. Blood was sampled before injection and 7 days after the first and fourth injection. Plasma was obtained by centrifugation at 1000 × g for 10 min. mPEG2000-DSPE and SP-PEG3400-DSPE dissolved in methanol was coated in 96-well microplate (2 μg/well). Plasma with different dilution by control plasma was incubated with coated plate. HRP conjugated anti-mouse IgG (ZB-2305, 1:5000) or IgM antibody (ab97230, 1:5000) was added and incubated for 1 h at room temperature. After three washes with PBS, TMB kit colorimetric measurement was used to detect the IgG and IgM produced in mouse plasma.

To evaluate inflammation response, BABL/c mice were injected through the tail vein isotonic sucrose, lipopolysaccharide (LPS, 10 μg kg^−1^) plus D-galactosamine (100 mg kg^−1^), sLip and SP-sLip (50 mg phospholipid per kg mice body weigh). Plasma was collected 6 h after injection and IL6 level was measured using ELISA kit according to the manufacturer’s manual.

### Evaluation of neurotoxicity

PC12 cells (1 × 10^4^ per mL) were seeded in 96-well plate in DMEM (10% FBS) and cultured overnight. Different concentrations of SP peptide were added into the culture for 48 h. MTS kit was used to assess cell viability.

Saline, DOX, sLip/DOX, and SP-sLip/DOX were administered through the tail vein of BALB/c mice at a total doxorubicin dose of 10 mg kg^−1^ for five times (at day 1, 3, 5, 7, and 9). At the day 10, mice were killed and organs were fixed with 4% PFA and sectioned. Slices were stained with H&E for biosafety evaluation.

### Statistical analysis

Data were presented as means ± SDs from the sample numbers (*N*). Data from experiments were analyzed with GraphPad Prism 6.0. A two-sided Student’s *t*-test with unpaired comparisons was used to evaluate differences in comparison of two groups. Levels of significance were set at *p* < 0.05. For quantitative analysis in SDS-PAGE and Western Blotting, values were calculated with the threshold and intensity of bands. Image J software (National Institutes of Health) was used for densitometric analysis, with each experimental sample normalized to sLip control, each *N* represents data collected from one independent experiment for protein corona isolation. For all the immunofluorescence staining, representative images from three animal tissues were calculated with signal area percentage via Image Pro software.

### Reporting summary

Further information on experimental design is available in the Nature Research Reporting Summary linked to this paper.

## Supplementary information

Supplementary Information

Reporting summary

## Data Availability

The data that support the findings of this study are available within the paper and the supplementary information. All other data are available from the authors upon reasonable request.
