# Supplementary material for: Brain-targeted drug delivery by manipulating protein corona functions
Source: Nat Commun. 2019 Aug 8;10:3561. doi: 10.1038/s41467-019-11593-z (PMC6687821; doi:10.1038/s41467-019-11593-z)
Supplement: Supplementary file 1 — Supplementary Information [file 41467_2019_11593_MOESM1_ESM.pdf]

Supplementary Information

Brain-targeted Drug Delivery by Manipulating Protein Corona Functions

Zhang et al

Supplementary Tables

Supplementary Table 1. Characterization of liposomes and PLGA nanoparticles. Data are means ± SD (n = 3).

	sLip	SP-sLip	PLGA NP	SP-PLGA NP
Size (nm)	157 ± 0.6	152 ± 2.0	121 ± 1.1	140 ± 1.8
PDI	0.02 ± 0.01	0.06 ± 0.02	0.08 ± 0.01	0.05 ± 0.01
Zeta-potential (mV)	-62.4 ± 0.7	-39.5± 2.5	-48.4 ± 1.6	-27.2± 0.5

Supplementary Figures

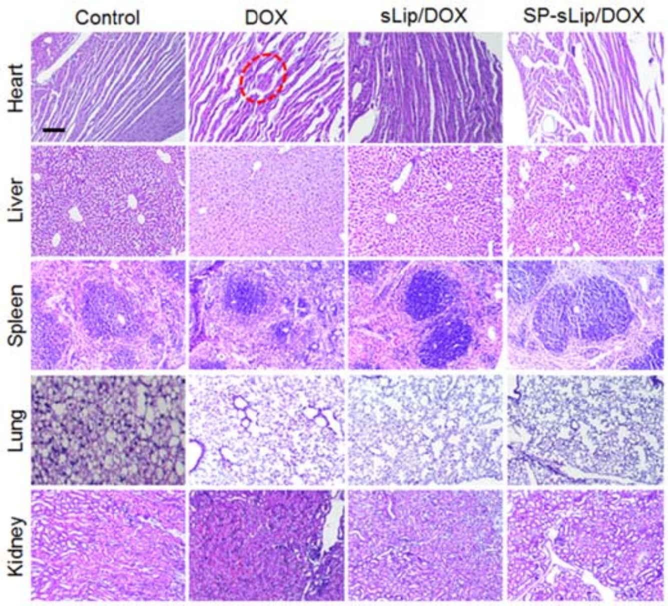

Supplementary Figure 1. Toxicity evaluation. Representative light microscopy of hematoxylin & eosin-stained sections of BALB/c mice main organs after five intravenous injections of saline, doxorubicin (DOX), sLip/DOX and SP-sLip/DOX (at a total doxorubicin dose of 10 mg kg^{-1}). Myocardial toxicity induced by doxorubicin was circled with dashed lines. Scale bar = 100 µm.

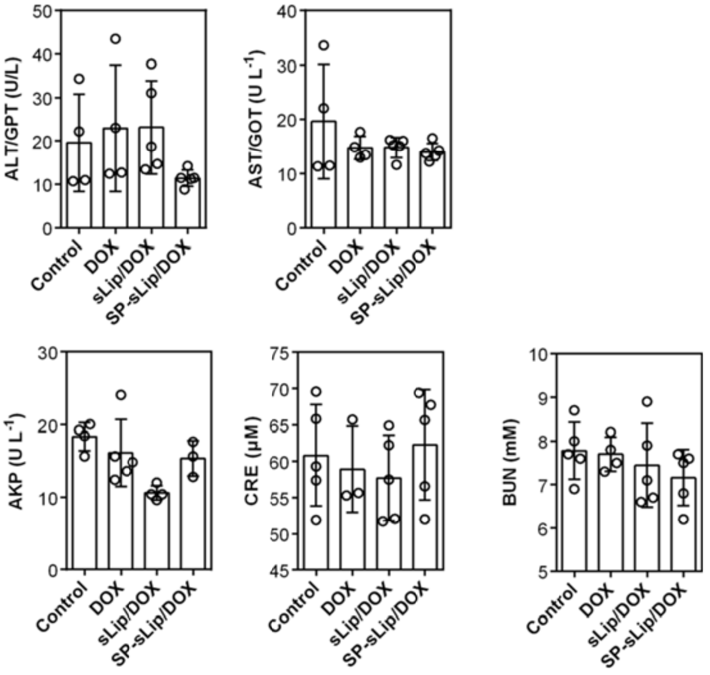

Supplementary Figure 2. Liver functions assessment. ALT/GTP (Alanine aminotransferase), AST/GOT (Aspartate aminotransferase), AKP (Alkaline phosphatase kinase), CRE (Creatinine), BUN (Blood Urea Nitrogen) were measured via related ELISA kits after five injections (every two days) of saline (Control), doxorubicin (DOX), doxorubicin loaded plain liposomes (sLip/DOX) and SP-sLip (SP-sLip/DOX) at a total doxorubicin dose of 10 mg kg^{-1}. Data are means ± SD (n = 3-5).

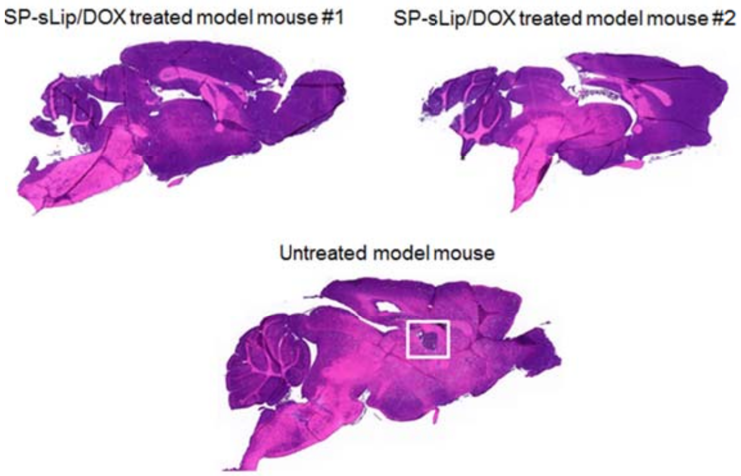

Supplementary Figure 3. Intracranial glioma evaluation. Microscopic observation of hematoxylin/eosin-stained brain sections of two survived nude mice after receiving SP-sLip/DOX treatment at 100 days after glioma implantation and untreated model mouse at 18 days after glioma implantation. Intracranial glioma was indicated by white square.

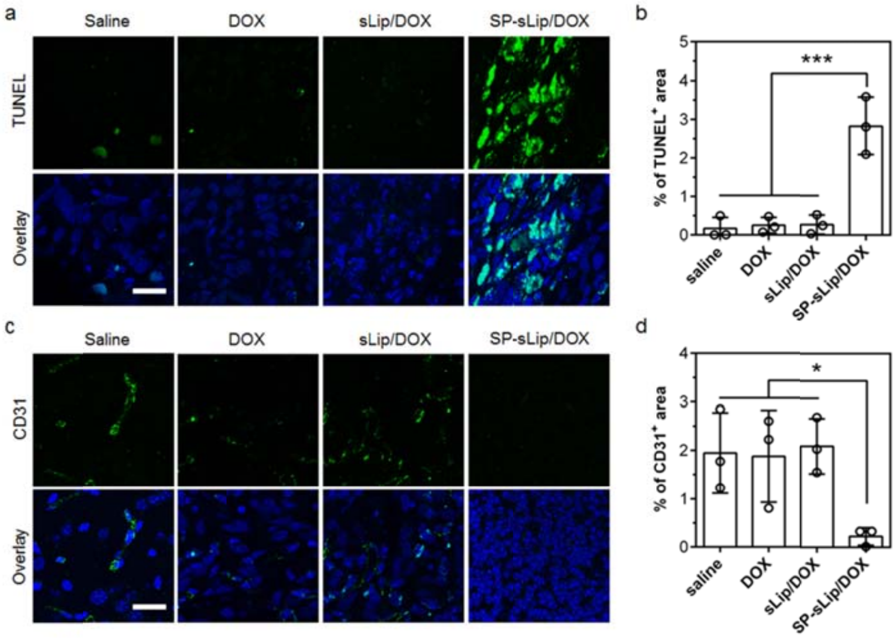

Supplementary Figure 4. Mechanistic study of anti-glioma effect of SP-sLip/DOX. (a) Effect of SP-sLip/DOX on *in vivo* intracranial glioma cell apoptosis. Nude mice bearing intracranial glioma cells received different DOX formulations (at a DOX dose of 2 mg kg^{-1}, five injections at day 7, 9, 11, 13, and 15 after glioma implantation) and were sacrificed at day 16. Brains were dissected and stained with DAPI (blue) and TUNEL kits (green). (b) Positive TUNEL staining and glioma cells in three random areas were counted by Image Pro. (c) Anti-angiogenic effect of SP-sLip/DOX. Brains dissected in (a) were stained with DAPI (blue) and anti-CD31 antibody (green). (d) Positive CD31 staining and glioma cells in brains from three mice were counted by Image Pro. Scale bar = 50 μm. Data are means ± SD (n = 3). *$p < 0.05$ and *** $p<0.001$ by student's t-test.

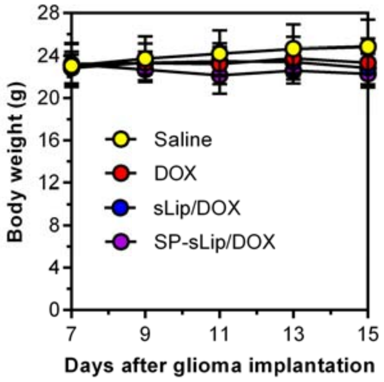

Supplementary Figure 5. Body weight of nude mice after receiving saline and different DOX formulations. Data are means ± SD (n = 16).

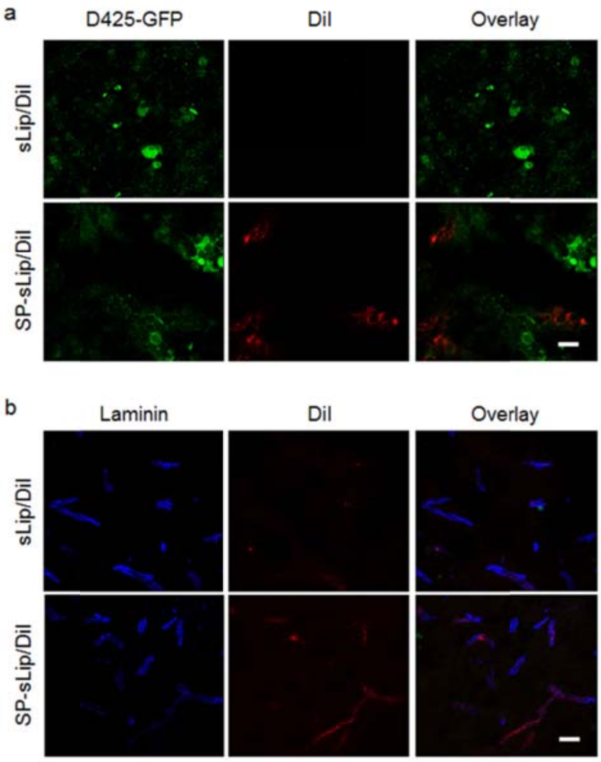

Supplementary Figure 6. Biodistribution of sLip and SP-sLip in the brain of nude mice bearing D425 (GFP labeled). DiI labeled sLip (sLip/DiI) and SP-sLip (SP-sLip/DiI) were injected via tail vein of nude mice bearing intracranial medulloblastoma (at day 18 after implantation). Mouse brains were dissected and sectioned at 4 h after administration. The tumor region (a) was directly observed (green, D425 cells; red, DiI) by confocal laser scanning microscopy. The non-tumor region (b) were stained with anti-laminin antibody (blue) before microscopic observation. Scale bar = 20 μm.

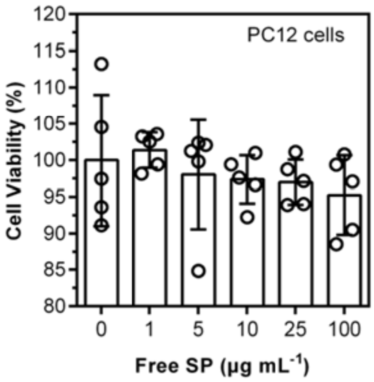

Supplementary Figure 7. Cytotoxicity of free SP peptide against PC12 cells after 48 h incubation at 37 °C. Data are means ± SD (n = 5).

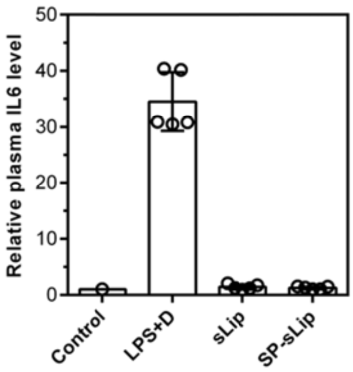

Supplementary Figure 8. Evaluation of pro-inflammatory cytokine response. Plasma IL6 level at 6 h after intravenous injection of isotonic sucrose (Control, negative control), lipopolysaccharide plus D-galactosamine (LPS+D, positive control), sLip or SP-sLip (50 mg phospholipid per kg mice body weight). Value of negative control was set as 1. Data are means ± SD (n = 5).

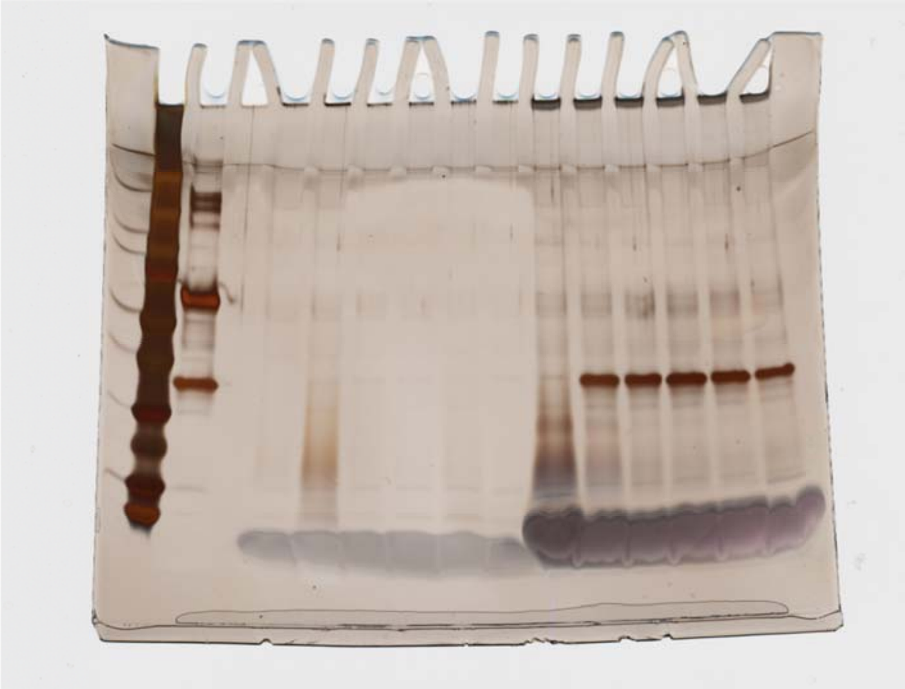

Supplementary Figure 9. The uncropped and unprocessed scan of Figure 1a.

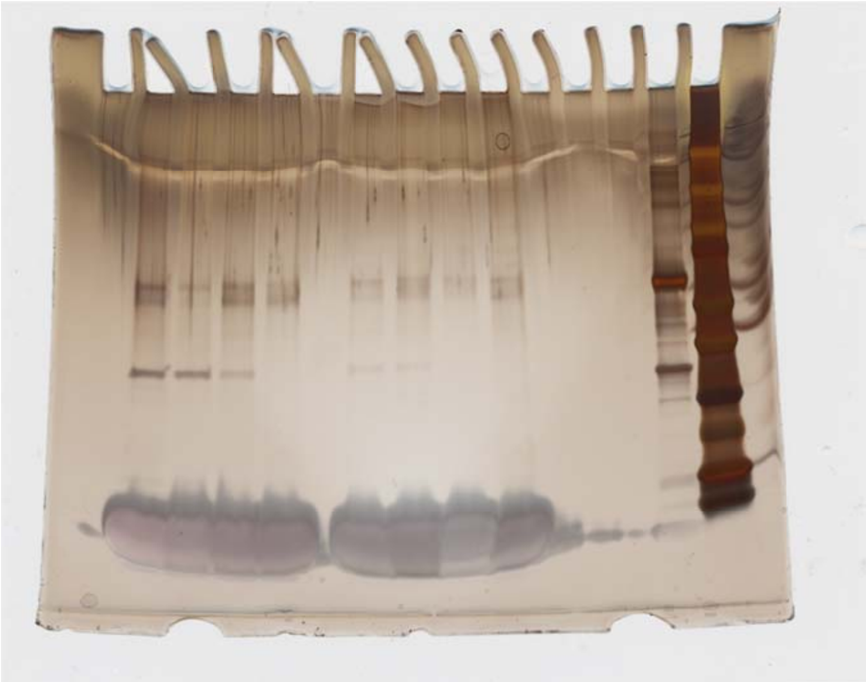

Supplementary Figure 10. The uncropped and unprocessed scan of Figure 1c.

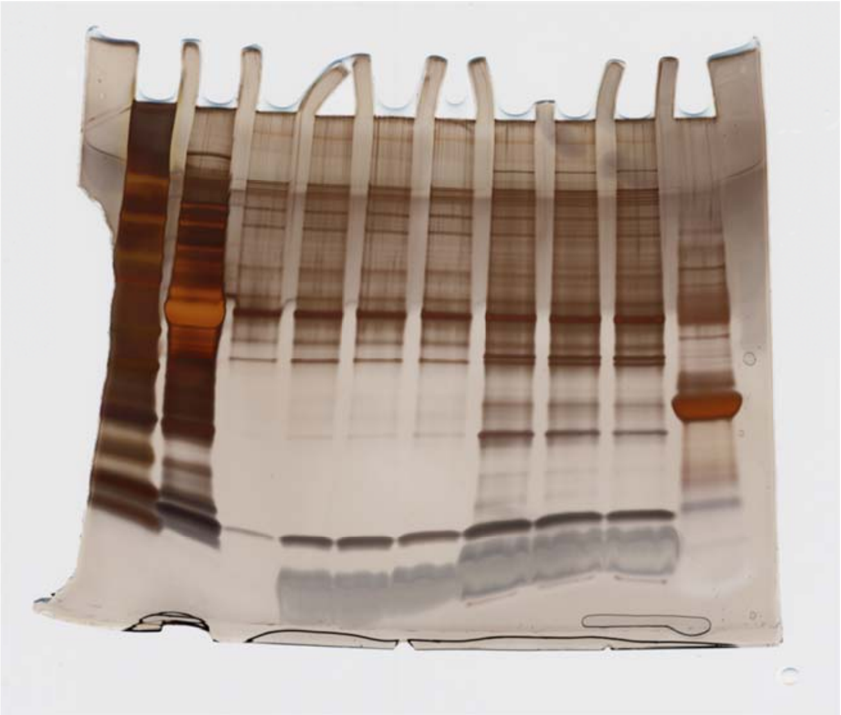

Supplementary Figure 11. The uncropped and unprocessed scan of Figure 2a.

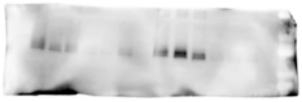

Supplementary Figure 12. The uncropped and unprocessed scan of ApoE in Figure 2c.

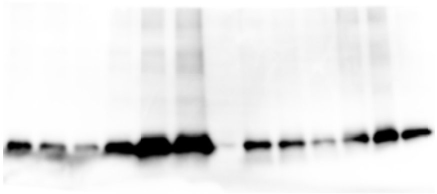

Supplementary Figure 13. The uncropped and unprocessed scan of ApoA1 in Figure 2c.

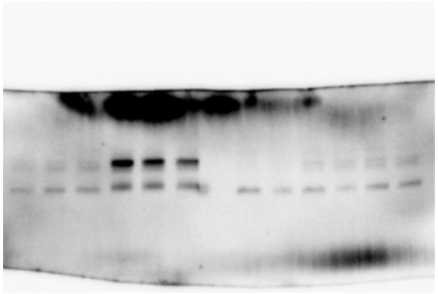

Supplementary Figure 14. The uncropped and unprocessed scan of ApoJ in Figure 2c.

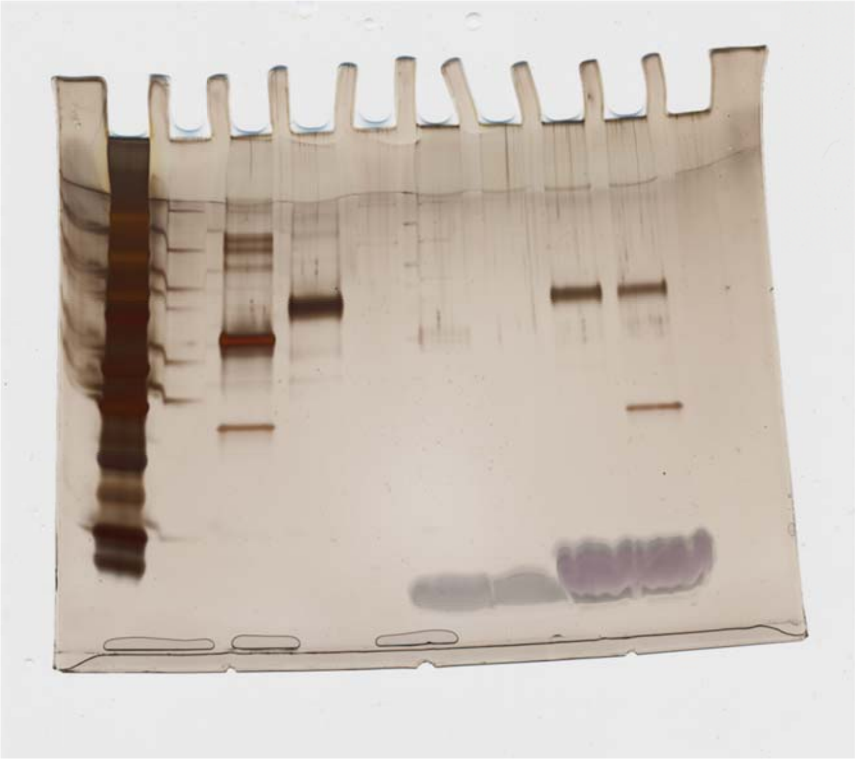

Supplementary Figure 15. The uncropped and unprocessed scan of Figure 3a.
